# Association between subjective well-being trajectories and anxiety/depression: findings from a nationally representative cohort study

**DOI:** 10.3389/fpsyg.2025.1573260

**Published:** 2025-06-27

**Authors:** Xinyu Wang, Qing Ke, Pengqun Leng, Dan Liu, Wen Zeng

**Affiliations:** ^1^Nursing School of Guizhou University of Traditional Chinese Medicine, Guiyang, China; ^2^Guizhou Provincial People’s Hospital, Guiyang, Guizhou, China; ^3^School of Nursing, Peking University Health Science Center, Beijing, China

**Keywords:** subjective well-being, cohort study, trajectories, anxiety, depression

## Abstract

**Background:**

Subjective well-being (SWB) in older adults is a key indicator of psychological health and quality of life. However, research on trajectories of SWB is quite limited, and little is known about the relationship between anxiety/depression and trajectories of SWB in older adults. This study aimed to identify the trajectories of SWB in older adults and examine the association between anxiety/depression and trajectories of SWB.

**Methods:**

A population-based cohort study, the National Health and Aging Trends Study in the United States from 2015 to 2022. SWB was assessed through 11 items, including positive/negative emotions (4 items), self-realization (4 items), self-efficacy, and resilience (3 items). Anxiety/depression symptoms were assessed by the Patient Health Questionnaire-4 (PHQ-4). The group-based trajectory modeling identified trajectories. The design-based weighted multinomial logistic regression was used to examine the association between anxiety/depression and SWB trajectories.

**Results:**

Of 5,057 included older adults, 59.22% were aged between 60 and 74 years old, 55.5% were female, and 82.23% were non-Hispanic White. Four trajectories of SWB were identified: Group 1 (*low, slightly-declining SWB*, 8.7%), Group 2 (*moderate, declining SWB*, 25.4%), Group 3 (*moderate, slightly-declining SWB*, 42.2%), and Group 4 (*high, slightly-declining SWB*, 23.7%). Higher anxiety/depression scores were associated with low or moderate SWB trajectories: Group 1 (odds ratio, OR: 3.35, 95% confidence interval, CI: 2.73, 4.00), Group 2 (OR: 2.59, 95% CI: 2.20, 3.00), and Group 3 (OR: 1.80, 95% CI: 1.49, 2.18).

**Conclusion:**

The trajectories of SWB varied among older adults. Anxiety/depression was significantly associated with SWB trajectories. Evidence-based effective strategies for the management of anxiety/depression are needed to maintain SWB at a high level in older adults.

**What does this paper contribute to the broader global clinical community?:**

• This study identifies four distinct trajectories of subjective well-being in older adults.
• Anxiety/depression is significantly associated with subjective well-being trajectories.
• Our study highlights the importance of screening and longitudinally tracking SWB in older adults.
• Effective prevention and intervention of anxiety/depression is a high priority to maintain or improve SWB in older adults.

## Introduction

As fertility rates continued to decline and life expectancy increased, global population aging became a pressing public health issue (Kanasi et al., 2016). Epidemiological studies indicated that 11% of the global population was over 60 years old, and this proportion was projected to reach 22% by 2050 (Newgard and Sharpless, 2013). In response to this trend, the World Health Organization (WHO) introduced the concept of “healthy ageing” in 1990, and in its 2015 *Global Report on Ageing and Health*, defined it as “the process of developing and maintaining the functional capabilities that enable older people to live well” (Beard et al., 2016, p. 29). Within this framework, maintaining physical and mental well-being was essential to improving quality of life and promoting active, inclusive aging.

Subjective well-being (SWB), as a key psychological indicator of life quality, has received increasing attention in aging research. SWB encompasses both cognitive evaluations (e.g., life satisfaction) and emotional experiences (e.g., positive and negative affect) (Diener et al., 1999), and was widely considered a core marker of psychological health and adaptive functioning in older adults (Jopp et al., 2015; Toh et al., 2020). Cross-sectional studies showed that SWB was associated with a range of sociodemographic and health-related factors, including gender, age, marital status, physical health, education, and economic status (Lee, 2022; Martín-María et al., 2023; Upenieks and Liu, 2022). Notably, these studies consistently identified a negative correlation between SWB and anxiety (Ventura-León et al., 2022), with anxiety being associated with lower life satisfaction and heightened negative emotions (Malone and Wachholtz, 2018).

However, because SWB was inherently dynamic—shaped by life events, health fluctuations, and social changes over time (Luhmann, 2017; Steinmayr et al., 2019)—cross-sectional studies, which captured only a snapshot at a single time point, were insufficient to uncover its developmental patterns. They failed to reveal intra-individual changes and the evolving nature of SWB in later life. To overcome these limitations, a longitudinal approach was warranted to systematically investigate how SWB changed over time and how it might have been influenced by psychological factors such as anxiety/depression.

Group-based trajectory modeling (GBTM), a statistical method based on finite mixture modeling, offered a robust analytical framework for uncovering distinct developmental trajectories within a heterogeneous population (Choi et al., 2012). Developed by Nagin and colleagues, GBTM allowed for the identification of subgroups of individuals who followed similar temporal patterns, thereby providing a nuanced understanding of how psychological constructs evolved with age (Nagin and Odgers, 2010). Unlike traditional longitudinal analyses, which relied heavily on predefined group classifications, GBTM empirically derived trajectory groups directly from the data, enhancing its capacity to detect naturally occurring patterns and test hypothesized pathways (Nagin and Odgers, 2010, p. 2).

Given these advantages, GBTM was particularly suited to examining the temporal dynamics of SWB in older adults. It revealed how individuals differed in their longitudinal experiences of well-being, and whether psychological risk factors such as anxiety/depression contributed to these divergent patterns.

Therefore, drawing on nationally representative longitudinal data, this study aimed to (1) identify distinct trajectories of SWB in older adults using GBTM and (2) examine how anxiety/depression levels were associated with these developmental trajectories.

## Materials and methods

### Data source and study population

The data used in this study were derived from the National Health and Aging Trends Study (NHATS), a nationally representative longitudinal study designed to provide an in-depth understanding of the health, functional status, disability, and long-term care needs of older adults in the United States. NHATS was launched in 2011 (Round 1), enrolling approximately 8,245 individuals aged 65 and older, who have since been followed annually. In 2015 (Round 5), a supplemental sample was added to maintain national representativeness.

To obtain a nationally representative sample of older adults, NHATS employed a multi-stage stratified sampling method. In the first stage, counties (or county equivalents) were used as the sampling units, from which Primary Sampling Units (PSUs) were selected nationwide. In the second stage, address-based sampling was conducted within these PSUs to ensure coverage across various racial/ethnic groups and geographic regions. In the third stage, eligible older adults were identified from the sampled addresses and selected for individual interviews.

Data were collected annually through face-to-face interviews, with response rates ranging from 71.6 to 96% (Liu et al., 2024). The data for each year were weighted to ensure national representativeness. NHATS provided weight variables for each survey round, including cross-sectional and longitudinal weights. These weights were adjusted to account for differences in sampling probabilities (e.g., oversampling of the oldest-old), non-response bias, and alignment with census data. These methodological adjustments ensure that the survey results statistically represent the characteristics of the U.S. population aged 65 and over.

This current study used data from Round 5 (2015) to Round 12 (2022) for analysis. The inclusion criteria were that SWB scores were without missing values in Round 5, Round 6, and Round 7. Finally, a total of 5,057 older adults were included in our study.

### Subjective well-being assessment

In this study, we assessed SWB through three domains, including positive/negative emotion (four items), self-realization (four items), and self-efficacy and resilience (three items). The total scores for SWB range from 11 to 41 points, with higher scores reflecting a higher level of SWB (Shown in Supplementary Table S1).

### Anxiety/depression

The Patient Health Questionnaire-4 (PHQ-4), with a Cronbach’s alpha coefficient of 0.82 (Meidl et al., 2024), was used to assess anxiety/depression in older adults (Freedman et al., 2022). The total scores range from 0 to 12, with higher scores reflecting a more severe level of anxiety/depression.

### Covariates

Covariates in this study include sociodemographic factors (e.g., age, gender, race, marital status, health status, education level, and annual income); lifestyle factors (e.g., body mass index (BMI) and smoking status); and clinical symptoms (e.g., hearing impairment, pain, speech problems, visual problems, swallowing problems, and breathing problems).

### Statistical analysis

We used Stata 17.0 (Stata Corp.) for the whole analysis. First, we used GBTM, developed by Nagin (2005), to identify the potential developmental trajectories of SWB among older adults. Considering model parsimony and clinical interpretability, the model shapes were fitted from two trajectory groups to five trajectory groups, with the polynomial function of time defined from cubic, square, to linear. After the modeling, model fit and classification accuracy were the core criteria for selecting the final model. We chose the Bayesian Information Criterion (BIC) as the preferred standard for evaluating group-based trajectory models because it balances model fit with complexity control, is applicable across various sample settings, and provides objective statistical support when constructing model structures based on theoretical considerations. Additionally, we used the Average Posterior Probability (AvePP) as an important basis for assessing the appropriateness of the trajectory model, as it allows for an objective evaluation of classification reliability and discriminative power in the absence of known true group membership. Furthermore, AvePP offers a clear empirical benchmark (e.g., ≥ 0.7), which effectively aids in both model selection and validation. The advantage of OCC (Odds of Correct Classification) lies in its ability to reflect not only classification accuracy but also the degree of improvement relative to random assignment, thereby offering stronger discriminative validity. When OCC ≥ 5.0, it indicates that the model has a high level of classification reliability; a value close to one suggests that the classification performance is nearly random and lacks practical utility. Therefore, using OCC as a diagnostic statistic for model selection helps assess the stability and reliability of the classification structure, aiding in the determination of the optimal number of trajectory groups and model scheme (Nagin, 2005). Based on Nagin’s suggestions, the following criteria were used to determine the final trajectory shapes: (1) BIC approaching 0; (2) AvePP ≥ 0.7; and (3) OCC > 5. After the SWB trajectories were identified, they were named according to their shapes and identified using a database statistical technique.

We accounted for the complex sampling designs during the analysis, including stratified sampling, primary sampling units, and weights. Weighted proportions described the distribution of categorical variables, and the distribution of continuous variables was presented by weighted medians (with standard errors, SE). Multinomial logistic regression was employed to analyze the association between anxiety/depression and SWB trajectories. The statistical significance was set at *P* < 0.05.

## Results

### Characteristics of the participants

This study included 5,057 participants in total (shown in Supplementary Figure S1). Among the participants, 59.22% were aged 60–74 years, 55.5% were female, and 82.23% were non-Hispanic White. SWB scores ranged from 35 (0.052)in 2015 to 35 (0.083) in 2022 (Shown in Supplementary Table S2). More details are displayed in Table 1.

**TABLE 1:** Characteristics and distribution differences across subgroups.

Characteristics	Total	Group 1	Group 2	Group 3	Group 4	*P*
	5,057	(8.7)	(25.4)	(42.2)	(23.7)	
**Anxiety/depression, median, (SE)**	1(0.041)	4(0.286)	3(0.120)	1(0.060)	0(0.039)	<0.001
**Age, (%)**						
60–74 years	59.22	4.11	11.58	26.88	16.64	<0.001
75–84 years	31.51	2.78	8.15	13.86	6.72	
85 + years	9.28	1.29	3.07	3.49	1.43	
**Gender, (%)**						
Male	44.50	37.52	41.61	44.9	48.76	0.007
Female	55.50	62.48	58.39	55.1	51.24	
**Race, (%)**						
Non-Hispanic white	82.23	80.91	80.89	83.14	82.23	0.622
Non-Hispanic black	8.12	7.32	8.55	8.01	8.19	
Other	3.08	2.37	3.59	3.11	2.8	
Hispanic	6.57	9.4	6.97	6.24	5.87	
**Marital status, (%)**						
Partnered	58.27	44.63	50.35	61.29	64.66	<0.001
Not partnered	41.73	55.37	49.65	38.71	35.34	
**Level of education, (%)**						
College degree	31.24	18.91	21.74	41.34	31.24	<0.001
level	29.30	20.72	29.21	30.04	30.89	
High school	24.68	30.83	30.00	24.41	18.26	
Less than high school	14.78	29.53	19.05	12.81	9.50	
**Annual total income, (%)**						
>$60,000	37.00	18.43	24.22	39.93	49.67	<0.001
$45,000–60,000	12.43	9.38	11.94	12.6	13.56	
$30,000–49,999	15.87	13.96	17.29	16.05	14.88	
$15,000–29,999	20.84	29.21	27.45	19.44	14,51	
< $15,000	13.86	29.03	19.11	11.98	7.37	
**BMI,%**						
Normal/non-obesity (<30)	67.37	61.96	63.24	68.69	70.51	0.008
Obesity (≥30)	32.63	38.04	36.76	31.31	29.49	
**Self-rated health, (%)**						
Good	81.52	46.40	68.33	87.87	93.90	<0.001
Fair	15.15	35.85	26.33	10.86	5.69	
Poor	3.33	17.75	5.34	1.27	0.41	
**Smoke, (%)**						
Yes	14.86	19.63	18.69	13.13	11.95	0.006
No	85.14	80.37	81.31	86.87	88.05	
**Hearing impairment, (%)**						
Yes	13.29	11.77	14.51	12.7	13.74	0.472
No	86.71	88.23	85.49	87.3	86.26	
**Visual impairment, (%)**						
Yes	62.83	67.13	60.95	63.31	62.27	0.259
No	37.17	32.87	39.05	36.69	37.73	
**Swallowing impairment, (%)**						
Yes	7.98	10.25	6.69	2.38	1.60	<0.001
No	92.02	89.75	93.31	97.62	98.40	
**Speech impairment, (%)**						
Yes	3.81	10.23	6.69	1.85	1.65	<0.001
No	96.19	89.77	93.71	98.15	98.35	
**Pain, (%)**						
Yes	53.33	76.82	64.51	52.32	37.12	<0.001
No	46.67	23.18	35.49	47.68	62.88	
**Breathing problems, (%)**						
Yes	18.24	40.67	26.66	14.35	10.06	<0.001
No	81.76	59.33	73.34	85.65	89.94	

### SWB trajectories

**FIGURE 1:**
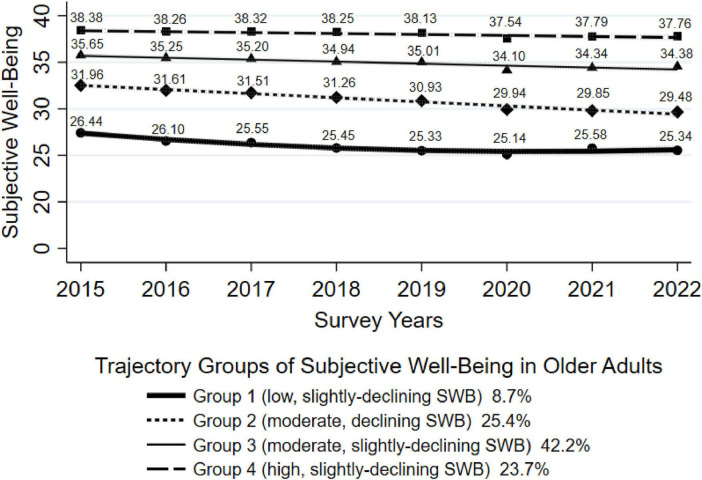
Trajectory groups of subjective well-being in older adults.

Using the statistics from the GBTM, we identified four trajectories of SWB, namely, Group 1 (*low, slightly-declining SWB*), Group 2 (*moderate, declining SWB*), Group 3 (*moderate, slightly-declining SWB*), and Group 4 (*high, slightly-declining SWB*), accounting for 8.7, 25.4, 42.2, and 23.7% of the included participants, respectively. Each of the four trajectory groups reflects the pattern of SWB scores over time as determined by the model. In Group 1, older adults had low SWB scores at baseline but slightly declined over time. In Group 2, older adults had moderate SWB scores at baseline, but decreased over time; In Group 3, older adults had moderate SWB scores at baseline, with a slight decline over time; and in Group 4, participants got high SWB scores at baseline, but slightly declined over time (shown in Figure 1). The fitting parameters in Supplementary Table S3 indicate a good model fit.

### Association between anxiety/depression and SWB trajectories

Compared to those in Group 4, participants in Group 1, Group 2, and Group 3 were more likely to have higher anxiety/depression scores (*P* < 0.01). When analyzing the association between anxiety/depression and the trajectory groups, we used a weighted multinomial logistic regression model. We found that anxiety/depression was strongly associated with SWB trajectories. In specific, when using participants in Group 4 as a reference, participants with higher anxiety/depression scores were more likely to be categorized into Group 1 (odds ratio, OR:3.35, 95% confidence interval (CI):2.73, 4.09), Group 2 (OR:2.59, 95% CI:2.20, 3.06), and Group 3 (OR:1.80, 95% CI:1.49, 2.18) after adjustment for all covariates (shown in Table 2).

**TABLE 2:** Weighted estimates of adjusted or in logistic regression of factors associated with subjective well-being in trajectory group membership (ref. group 4).

Subgroups	Group1			Group2			Group3		
	RRR	(95% CI)	*P*-value	RRR	(95% CI)	*P*-value	RRR	(95% CI)	*P-*value
Anxiety/depression	3.35	(2.73–4.09)	< 0.001	2.59	(2.20–3.06)	< 0.001	1.80	(1.49–2.18)	< 0.001
**Age, years (reference: 60–74)**									
75–84	1.63	(0.81–3.25)	0.166	1.49	(1.03–2.15)	0.034	1.12	(0.82–1.53)	0.479
85 +	1.09	(0.26–4.57)	0.908	1.90	(0.86–4.17)	0.109	1.02	(0.61–1.69)	0.951
**Sex (reference: male)**									
Female	0.40	(0.17–0.98)	0.044	0.52	(0.35–0.78)	0.002	0.70	(0.50–0.98)	0.037
**Race and ethnicity (reference: non-Hispanic white)**									
Non-Hispanic black	0.23	(0.09–0.60)	0.003	0.51	(0.33–0.81)	0.005	0.81	(0.52–1.27)	0.346
Other	0.00	(0.00–0.00)	< 0.001	3.58	(0.83–15.40)	0.086	2.92	(0.54–15.85)	0.209
Hispanic	0.78	(0.24–2.54)	0.681	0.37	(0.16–0.89)	0.027	0.60	(0.25–1.47)	0.258
**Marital status (reference: partnered)**									
Not partnered	2.07	(0.87–4.93)	0.099	0.96	(0.58–1.60)	0.874	1.12	(0.76–1.63)	0.565
**Education status (reference: less than a college degree)**									
Diploma level	0.36	(0.14–0.90)	0.030	0.90	(0.54–1.49)	0.673	1.09	(0.72–1.65)	0.682
High school	1.02	(0.38–2.73)	0.972	1.99	(1.18–3.36)	0.011	1.34	(0.83–2.15)	0.222
Less than high school	1.65	(0.47–5.77)	0.427	1.39	(0.70–2.77)	0.344	1.01	(0.54–1.87)	0.986
**Annual income, $ (reference: > 60,000)**									
45,000–60,000	0.51	(0.11–2.49)	0.401	1.39	(0.68–2.83)	0.362	0.77	(0.50–1.21)	0.254
30,000–44,999	1.79	(0.65–4.92)	0.252	1.68	(0.86–3.29)	0.126	0.98	(0.54–1.78)	0.945
15,000–29,999	1.81	(0.55–6.00)	0.323	1.67	(0.83–3.38)	0.148	1.29	(0.71–2.35)	0.398
<15,000	2.57	(0.67–9.91)	0.166	1.89	(0.72–4.92)	0.190	1.78	(0.87–3.63)	0.114
**BMI (reference: normal/non-obesity)**									
Obesity	0.87	(0.39–1.94)	0.731	1.21	(0.75–1.94)	0.425	0.90	(0.60–1.36)	0.620
**Self-rated health (reference: good)**									
Fair	5.31	(1.97–14.32)	0.001	4.13	(2.18–7.81)	< 0.001	1.53	(0.86–2.73)	0.240
Poor	7.77	(0.66–91.81)	0.102	5.99	(0.82–43.57)	0.076	2.19	(0.31–15.68)	0.340
**Smoke (reference: yes)**									
No	0.42	(0.15–1.13)	0.083	0.68	(0.39–1.17)	0.159	1.00	(0.59–1.69)	0.991
**Hearing impairment (reference: no)**									
Yes	1.08	(0.29–4.06)	0.903	1.08	(0.61–1.91)	0.792	0.97	(0.64–1.49)	0.898
**Visual impairment (reference: no)**									
Yes	1.98	(0.75–5.25)	0.166	0.99	(0.63–1.54)	0.962	1.13	(0.79–1.60)	0.505
**Swallowing impairment (reference: no)**									
Yes	2.09	(0.57–7.63)	0.260	1.66	(0.61–4.55)	0.319	1.84	(0.68–5.00)	0.228
**Speech impairment (reference: no)**									
Yes	1.71	(0.50–5.79)	0.384	1.08	(0.38–3.11)	0.884	0.79	(0.28–2.19)	0.640
**Pain (reference: no)**									
Yes	6.78	(2.96–15.55)	< 0.001	2.59	(1.79–3.73)	< 0.001	1.81	(1.37–2.40)	< 0.001
**Breathing problems (reference: no)**									
Yes	1.47	(0.72–3.00)	0.285	1.26	(0.79–1.99)	0.320	0.94	(0.55–1.61)	0.819

## Discussion

Understanding the relationship between SWB and anxiety/depression among older adults was not straightforward, likely due to multiple complex factors. First, as individuals aged, they experienced various physical, psychological, and social changes that affected different individuals differently, resulting in significant heterogeneity in well-being trajectories. Although anxiety/depression was considered a common mental health issue among older adults, its dynamic interaction with SWB over time had not been sufficiently explored. Many existing studies relied on cross-sectional data, which limited the understanding of causal relationships and long-term change patterns. Additionally, cultural, social, and individual differences further complicated this relationship, as the experience of anxiety/depression and well-being varied across different populations and environments. Therefore, studying how anxiety influences the developmental trajectories of SWB in older adults was of great importance for identifying vulnerable groups, understanding underlying mechanisms, and developing targeted interventions to promote successful aging.

### Empirical findings: heterogeneous SWB trajectories

However, most previous studies of SWB in older adults have focused on cross-sectional designs, and the results generally showed that the overall SWB of older adults was not optimistic (Lee, 2021; Qin et al., 2024). However, cross-sectional studies have limitations and cannot track long-term changes in the SWB of older adults. Different from those cross-sectional studies, through a 7-year longitudinal cohort study utilizing the GBTM, our study identified four distinct patterns of SWB trajectories: group 1 (*low, slightly-declining SWB*), group 2 (*moderate, declining SWB*), group 3 (*moderate, slightly-declining SWB*), and group 4 (*high, slightly-declining SWB*). The dynamic characteristics of SWB in the elderly have been confirmed, and individual differences have been emphasized. Therefore, healthcare professionals should focus more on the long-term changes in elderly SWB, rather than just the health status at a single time. As a result, there is an urgent need for early screening, regular monitoring, and longitudinal tracking of SWB.

### Methodological considerations: use of GBTM

In terms of methodological choice, our study selected GBTM as the core analytical strategy, primarily due to its strong compatibility with our dataset’s characteristics and the study’s objectives. Unlike traditional growth curve modeling or repeated measures ANOVA, which typically assume that the overall sample follows a single average trajectory with individual deviations around it, GBTM is a data-driven, person-centered approach that identifies substantively distinct latent subgroups and characterizes their developmental trajectories over time. This method was particularly well-suited to the NHATS dataset, a large-scale, multi-wave, nationally representative longitudinal panel. Among older adults, the trajectories of SWB often exhibit substantial heterogeneity. By applying GBTM, we were able to effectively capture such variations, thereby overcoming the limitations of conventional methods. During the model selection process, we conducted a comprehensive theoretical and methodological assessment of GBTM’s appropriateness. We found that it not only provided a statistically valid modeling framework but also significantly enhanced the interpretability and practical value of our findings. This facilitated the precise identification of psychologically vulnerable subpopulations and provided a robust empirical basis for developing targeted interventions and policies aimed at promoting SWB among older adults. Thus, the application of GBTM in this study was methodologically justified and offered a novel perspective for understanding the diverse developmental trajectories of mental health in later life.

### Mechanisms linking anxiety/depression and SWB

Importantly, the study results indicate a significant negative correlation between anxiety/depression and the trajectory of SWB. Older adults with higher anxiety/depression scores are more likely to be classified into poorer trajectory groups. This finding could be explained through multiple interconnected physiological, psychological, and behavioral mechanisms. Firstly, anxiety/depression significantly impact the normal functioning of the hypothalamic-pituitary-adrenal (HPA) axis, leading to disruptions in cortisol secretion rhythms. This dysregulation of the neuroendocrine system triggers a range of typical physiological symptoms, including sleep disturbances, changes in appetite, and persistent fatigue (Kinlein et al., 2022). Notably, these physiological disruptions not only directly impair individuals’ daily functioning but also further diminish quality of life by depleting physical and mental reserves, ultimately leading to a sustained decline or severe fluctuations in SWB (Lenneis et al., 2024).

Secondly, anxiety/depression can significantly impair emotional regulation abilities, making them more vulnerable when facing daily stressors and challenges. This depletion of psychological resources leads to a reduction in positive life experiences and a continuous decline in SWB. This phenomenon is particularly pronounced among the elderly. Existing evidence suggests that anxiety/depression levels in older adults are significantly negatively correlated with self-efficacy (Luszczynska et al., 2005), which is an important psychological resource for SWB. Specifically, the higher the levels of anxiety/depression, the weaker the elderly’s confidence in perceiving and coping with challenging situations (Mateusz Cybulski et al., 2017). Current research provides a reasonable explanation for this phenomenon: anxiety/depression amplify individuals’ subjective perception of stress while simultaneously depleting their coping resources (Omran and Mcmillan, 2018). Moreover, as these psychological states persist, individuals may develop feelings of loneliness and helplessness, which further weaken self-efficacy, making them more prone to interpret life events negatively and to evaluate their quality of life poorly (Lee et al., 2023). A previous study also reported a significant inverse relationship between anxiety/depression and resilience. One study found that resilience was negatively correlated with depression (*r* = −0.39, *p* = 0.002) and anxiety (*r* = −0.27, *p* = 0.04) (Kutcher et al., 2023). In other words, the lower the resilience level, the harder it is for older adults to recover from life adversities, making them more likely to fall into a vicious cycle of negative emotions (Cybulski et al., 2017; Górska et al., 2022). The lack of resilience and self-efficacy, as psychological protective factors, contributes to an adverse development in the trajectory of SWB.

Finally, anxiety/depression not only affect older adults’ daily functioning, social participation, and overall quality of life (Han et al., 2020; Liu et al., 2023) but might also further influence their social functioning through behavioral pathways. These emotional states were often closely associated with social withdrawal, loss of interest, and reduced activity, weakening the protective function of the social support system among older adults, resulting in a vicious cycle of “emotional distress–social isolation,” ultimately exerting a profound negative impact on their SWB (Liao et al., 2022).

### Cultural considerations and social context

Our study speculated that this process might be more pronounced within specific cultural contexts. In the United States, the sociocultural environment strongly emphasizes individualism, independence, and the pursuit of self-worth (Markus and Kitayama, 2014). Many older adults tend to maintain an independent living style, which can enhance their sense of autonomy and control over life, aligning with the concept of “successful aging.” However, given the condition of declining health, the loss of a spouse, or physical limitations, living alone, older adults might be lacking in emotional support and experience increased feelings of loneliness. Compared to those living with others, older adults living independently are more susceptible to social isolation and psychological distress, thereby experiencing more pronounced negative effects of anxiety/depression on mental health (Cacioppo and Cacioppo, 2014). This structural vulnerability embedded within the cultural context may further exacerbate the decline in SWB and affect its long-term developmental trajectory in later life.

### Protective factors and stable high SWB trajectories

In contrast, older adults with lower anxiety/depression scores were more likely to maintain higher or more stable levels of SWB. Previous studies showed that life attitude, chronic disease management abilities, and the quality of social networks were significantly associated with higher level of well-being in older adults (Steptoe et al., 2015). Older adults in the “high but slightly declining well-being” trajectory exhibited more positive characteristics in terms of self-efficacy, social participation, and family support. They might possess a higher sense of self-efficacy, actively engage in regular physical exercise and various social activities, and maintain stable and close interpersonal relationships and social networks, thereby effectively mitigating the negative impacts of aging (Liu et al., 2016).

### Implications

The longitudinal assessment of SWB in older adults provides valuable insights with significant clinical and policy implications for promoting mental health and enhancing functional independence in later life. By identifying individuals in the “low decline” and “moderate decline” trajectories at an early stage, healthcare providers could implement timely and targeted interventions to mitigate the risk of further deterioration in SWB. Notably, this study highlights the significant impact of anxiety/depression symptoms on the trajectories of SWB, underscoring the necessity of incorporating mental health screening and management into routine geriatric care. Based on our findings, we suggest that community healthcare services implement regular psychological screening programs to detect anxiety/depression in older adults. Psychosocial interventions such as emotional regulation workshops, peer-support groups, and exercise-based therapies should be actively promoted to address emotional distress and maintain a high level of SWB over time in older adults. From a policy standpoint, strengthening the structure and accessibility of social support networks is equally essential, as these resources play a critical role in helping older adults cope with psychological stressors. Developing integrated, community- and home-based mental health service models can contribute to sustaining higher levels of SWB and enhancing the overall quality of life in the aging population.

### Limitations

This study had several limitations. First, it failed to collect key time-sensitive information, such as sudden changes in health status or major life events, which could have significantly impacted the trajectories of SWB. Second, although the study employed GBTM to identify four distinct SWB trajectories, GBTM, as a finite mixture model, assumed that each individual belonged exclusively to a specific trajectory group. This “hard classification” approach might have overlooked the potential fluidity of individuals across different trajectories. Furthermore, the model-fitting results largely depended on the researchers’ subjective decisions regarding the shape of the trajectories and the number of groups, which could have led to variations in the identified trajectories and affected the stability and generalizability of the conclusions. Future studies should enhance the dynamic monitoring and collection of time-sensitive events to better capture the key factors influencing changes in SWB. In addition, it is recommended that the findings be replicated in larger samples or across different cultural contexts to improve their external validity and practical applicability.

## Conclusion

In conclusion, this study is the first to identify the trajectories of SWB in older adults, and we found a significant association between anxiety/depression and the trajectories of SWB in older adults. This finding provides a theoretical basis for future mental health interventions. Particularly, our study highlights the importance of screening and longitudinally tracking SWB in older adults, as well as effective evidence-based strategies for the prevention of anxiety/depression to maintain or improve in older adults.

## Data Availability

The datasets presented in this study can be found in online repositories. The names of the repository/repositories and accession number(s) can be found in the article/Supplementary material.

## References

[B1] Beard J. R. Officer A. de Carvalho I. A. Sadana R. Pot A. M. Michel J. P. (2016). The World report on ageing and health: A policy framework for healthy ageing. *Lancet* 387 2145–2154. 10.1016/s0140-6736(15)00516-4 26520231 PMC4848186

[B2] Cacioppo J. T. Cacioppo S. (2014). Social relationships and health: The toxic effects of perceived social isolation. *Soc. Pers. Psychol. Compass* 8 58–72. 10.1111/spc3.12087 24839458 PMC4021390

[B3] Choi C. W. Stone R. A. Kim K. H. Ren D. Schulz R. Given C. W. (2012). Group-based trajectory modeling of caregiver psychological distress over time. *Ann. Behav. Med.* 44 73–84. 10.1007/s12160-012-9371-8 22585179 PMC3880564

[B4] Cybulski M. Cybulski L. Krajewska-Kulak E. Cwalina U. (2017). The level of emotion control, anxiety, and self-efficacy in the elderly in Bialystok, Poland. *Clin. Intervent. Aging* 12 305–314. 10.2147/cia.S128717 28223788 PMC5308481

[B5] Diener E. Suh E. M. Lucas R. E. Smith H. L. (1999). Subjective well-being: Three decades of progress. *Psychol. Bull.* 125 276–302. 10.1037/0033-2909.125.2.276

[B6] Freedman V. A. Schrack J. Skehan M. Kasper J. (2022). *National Health and Aging Trends Study User Guide: Rounds 1-11 Final Release.* Baltimore, MD: Johns Hopkins University School of Public Health.

[B7] Górska S. Singh Roy A. Whitehall L. Irvine Fitzpatrick L. Duffy N. Forsyth K. (2022). A systematic review and correlational meta-analysis of factors associated with resilience of normally aging, community-living older adults. *Gerontologist* 62 e520–e533. 10.1093/geront/gnab110 34346489 PMC9579466

[B8] Han K. Yang S. Jia W. Wang S. Song Y. Cao W. (2020). Health-related quality of life and its correlation with depression among chinese centenarians. *Front. Public Health* 8:580757. 10.3389/fpubh.2020.580757 33194985 PMC7661682

[B9] Jopp D. S. Wozniak D. Damarin A. K. De Feo M. Jung S. Jeswani S. (2015). How could lay perspectives on successful aging complement scientific theory? Findings from a US and a German life-span sample. *Gerontologist* 55 91–106. 10.1093/geront/gnu059 24958719 PMC5994883

[B10] Kanasi E. Ayilavarapu S. Jones J. (2016). The aging population: Demographics and the biology of aging. *Periodontology* 72 13–18. 10.1111/prd.12126 27501488

[B11] Kinlein S. A. Wallace N. K. Savenkova M. I. Karatsoreos I. N. (2022). Chronic hypothalamic-pituitary-adrenal axis disruption alters glutamate homeostasis and neural responses to stress in male C57Bl6/N mice. *Neurobiol. Stress* 19:100466. 10.1016/j.ynstr.2022.100466 35720261 PMC9198473

[B12] Kutcher A. Do Byon H. Esquivel J. (2023). Depression, anxiety and resilience: The association of emotions on self-care in patients with heart failure. *J. Cardiac. Fail.* 29:560. 10.1016/j.cardfail.2022.10.035 39137264

[B13] Lee J. W. Nersesian P. V. Suen J. J. Mensah Cudjoe T. K. Gill J. Szanton S. L. (2023). Loneliness is associated with lower coping self-efficacy among older adults. *J. Appl. Gerontol.* 42 270–279. 10.1177/07334648221129858 36178675 PMC9840677

[B14] Lee S. (2021). Social exclusion and subjective well-being among older adults in europe: Findings from the European social survey. *J. Gerontol. Series B* 76 425–434. 10.1093/geronb/gbaa172 33247758 PMC7813197

[B15] Lee S. (2022). Subjective well-being and mental health during the pandemic outbreak: Exploring the role of institutional trust. *Res. Aging* 44 10–21. 10.1177/0164027520975145 33234059

[B16] Lenneis A. Das-Friebel A. Tang N. K. Sanborn A. N. Lemola S. Singmann H. (2024). The influence of sleep on subjective well-being: An experience sampling study. *Emotion* 24:451. 10.1037/emo0001268. 37535565

[B17] Liao H. Liao S. Gao Y. J. Mu J. P. Wang X. Chen D. S. (2022). Correlation between sleep time, sleep quality, and emotional and cognitive function in the elderly. *Biomed. Res. Int.* 2022:9709536. 10.1155/2022/9709536 35607303 PMC9124129

[B18] Liu L. Gou Z. Zuo J. (2016). Social support mediates loneliness and depression in elderly people. *J. Health Psychol.* 21 750–758. 10.1177/1359105314536941 24925547

[B19] Liu P. Chen H. Tong B. Zhu D. Cong X. Shang S. (2024). Association between multisite musculoskeletal pain and disability trajectories among community-dwelling older adults. *Aging Clin. Exp. Res.* 36:115. 10.1007/s40520-024-02764-0 38780859 PMC11116213

[B20] Liu R. He W. B. Cao L. J. Wang L. Wei Q. (2023). Association between chronic disease and depression among older adults in China: The moderating role of social participation. *Public Health* 221 73–78. 10.1016/j.puhe.2023.06.003 37421756

[B21] Luhmann M. (2017). “The development of subjective well-being,” in *Personality development across the lifespan*, ed. Specht J. (Amsterdam: Elsevier), 197–218.

[B22] Luszczynska A. Gutiérrez-Doña B. Schwarzer R. (2005). General self-efficacy in various domains of human functioning: Evidence from five countries. *Int. J. Psychol.* 40 80–89. 10.1080/00207590444000041

[B23] Malone C. Wachholtz A. (2018). The relationship of anxiety and depression to subjective well-being in a Mainland Chinese sample. *J. Relig. Health* 57 266–278. 10.1007/s10943-017-0447-4 28702737 PMC5764815

[B24] Markus H. R. Kitayama S. (2014). “Culture and the self: Implications for cognition, emotion, and motivation,” in *College Student Development and Academic Life*, eds Altbach P. G. Arnold K. King I. C. (London: Routledge), 264–293.

[B25] Martín-María N. Lara E. Cabello M. Olaya B. Haro J. M. Miret M. (2023). To be happy and behave in a healthier way. A longitudinal study about gender differences in the older population. *Psychol. Health* 38 307–323. 10.1080/08870446.2021.1960988 34353185

[B26] Meidl V. Dallmann P. Leonhart R. Bretthauer B. Busch A. Kubosch E. J. (2024). Validation of the Patient Health Questionnaire-4 for longitudinal mental health evaluation in elite Para athletes. *PM&R* 16 141–149. 10.1002/pmrj.13011 37294844

[B27] Nagin D. (2005). *Group-Based Modeling of Development.* Cambridge, MA: Harvard University Press.

[B28] Nagin D. S. Odgers C. L. (2010). Group-based trajectory modeling in clinical research. *Annu. Rev. Clin. Psychol.* 6 109–138. 10.1146/annurev.clinpsy.121208.131413 20192788

[B29] Newgard C. B. Sharpless N. E. (2013). Coming of age: Molecular drivers of aging and therapeutic opportunities. *J. Clin. Investig.* 123 946–950. 10.1172/JCI68833 23454756 PMC3582156

[B30] Omran S. Mcmillan S. (2018). Symptom severity, anxiety, depression, self-efficacy and quality of life in patients with cancer. *Asian Pac. J. Cancer Prevent.* 19 365. 10.22034/APJCP.2018.19.2.365 29479979 PMC5980921

[B31] Qin A. Wu Y. Xin T. Xu L. Fu J. (2024). Lifestyle factors and subjective well-being among older adults in China: A national community-based cohort study. *Geriatr. Nurs.* 57 232–242. 10.1016/j.gerinurse.2024.04.018 38723544

[B32] Steinmayr R. Wirthwein L. Modler L. Barry M. M. (2019). Development of subjective well-being in adolescence. *Int. J. Environ. Res. Public Health* 16:3690. 10.3390/ijerph16193690 31575056 PMC6801746

[B33] Steptoe A. Deaton A. Stone A. A. (2015). Subjective well-being, health, and ageing. *Lancet* 385 640–648. 10.1016/S0140-6736(13)61489-0 25468152 PMC4339610

[B34] Toh W. X. Yang H. Hartanto A. (2020). Executive function and subjective well-being in middle and late adulthood. *J. Gerontol. Series B* 75 e69–e77. 10.1093/geronb/gbz006 30657966 PMC7457179

[B35] Upenieks L. Liu Y. (2022). Marital strain and support and subjective well-being in later life: Ascribing a role to childhood adversity. *J. Aging Health* 34 550–568. 10.1177/08982643211048664. 34666514

[B36] Ventura-León J. Caycho-Rodríguez T. Talledo-Sánchez K. Casiano-Valdivieso K. (2022). Depression, COVID-19 anxiety, subjective well-being, and academic performance in university students with COVID-19-infected relatives: A network analysis. *Front. Psychol.* 13:837606. 10.3389/fpsyg.2022.837606 35222215 PMC8867004

