# Supplementary material for: Association between subjective well-being trajectories and anxiety/depression: findings from a nationally representative cohort study
Source: Front Psychol. 2025 Jun 27;16:1573260. doi: 10.3389/fpsyg.2025.1573260 (PMC12248251; doi:10.3389/fpsyg.2025.1573260)
Supplement: Supplementary file 1 [file Table_1.docx]

Contents of the supplementary

[Flowchart of NHATS study population 1](#_Toc157177404)

[Table S1](#_Toc157177405) [Subjective well-being rating scale 2](#_Toc157177405)

[Table S2 The median and standard error of the subjective well-being scores for the years 2015 to 2022 (round 5 to round 12).](#_Toc157177408) 3

[Table S3 Number of identified trajectory groups and fitting parameters](#_Toc157177408) 4

[Table S4 Code of independent variables 8](#_Toc157177409)

#

# e-Flowchart of NHATS study population

Included in the NHATS cohort

(8334)

Exclusion due to not reaching 60 years of age (0)

((

Screened for eligibility

(8334)

Analyzed for trajectories

(5057)

Excluded because subjective well-being was not coded (3277)

((

Table S1 Subjective well-being rating scale

| Item | Question | Scoring Standard |
| --- | --- | --- |
| positive and negative emotions | feel cheerful | 1=Never, 2=rarely, 3=some days, 4=most days, 5=every day |
|  |  |  |
|  | feel bored | 1=every day, 2=most days, 3=some days, 4=rarely, 5=never |
|  |  |  |
|  | feel full of life | 1=Never, 2=rarely, 3=some days, 4=most days, 5=every day |
|  |  |  |
|  | feel upset | 1=every day, 2=most days, 3=some days, 4=rarely, 5=never |
|  |  |  |
| self-realization | life is meaningful | 1=agree not at all, 2=agree a little, 3=agree a lot |
|  |  |  |
|  | feel confident | 1=agree not at all, 2=agree a little, 3=agree a lot |
|  |  |  |
|  | gave up improving my life | 1=agree a lot, 2=agree a little, 3=agree not at all |
|  |  |  |
|  | like my living status | 1=agree not at all, 2=agree a little, 3=agree a lot |
|  |  |  |
| self-efficacy and resilience | determined by other people | 1=agree a lot, 2=agree a little, 3=agree not at all |
|  |  |  |
|  | easily find a way | 1=agree not at all, 2=agree a little, 3=agree a lot |
|  |  |  |
|  | easily adjusting to change | 1=agree not at all, 2=agree a little, 3=agree a lot |

Table S2 The median and standard error of the subjective well-being scores for the years 2015 to 2022 (round 5 to round 12).

|  | 2015（Round5） | 2016（Round6） | 2017（Round7） | 2018（Round8） | 2019（Round9） | 2020（Round10） | 2021（Round11） | 2022（Round12） |
| --- | --- | --- | --- | --- | --- | --- | --- | --- |
| SWB  Median,(SE) | 35 (0.052） | 35 (0.057) | 35 (0.059) | 35 (0.064) | 35 (0.067) | 34 (0.073) | 35 (0.076) | 35 (0.083) |

Table S3 Number of identified trajectory groups and fitting parameters

| Trajectory order | BIC1 | BIC2 | AIC | OCC1 | OCC2 | OCC3 | OCC4 | Entropy |
| --- | --- | --- | --- | --- | --- | --- | --- | --- |
| (2 2) | -86844.15 | -86836.65 | -86810.53 | 73.69664 | 56.57634 |  |  | 0.884 |
| (2 1) | -86841.66 | -86835.1 | -86812.25 | 73.72361 | 56.54626 |  |  | 0.884 |
| (2 0) | -86978.08 | -86972.45 | -86952.87 | 69.64249 | 55.83937 |  |  | 0.880 |
| (1 2) | -86845.01 | -86838.45 | -86815.6 | 74.59172 | 56.10682 |  |  | 0.884 |
| (1 1) | -86842.79 | 86837.16 | 86817.57 | 75.09452 | 55.7224 |  |  | 0.884 |
| (1 0) | -86979.26 | -86974.57 | -86958.25 | 70.39878 | 55.44715 |  |  | 0.881 |
| (0 2) | -87057.48 | -87051.85 | -87032.27 | 78.94883 | 54.50935 |  |  | 0.887 |
| (0 1) | -87056.28 | -87051.59 | -87035.26 | 79.31404 | 54.08142 |  |  | 0.887 |
| (0 0) | -87197.29 | -87193.54 | -87180.48 | 80.09494 | 49.29572 |  |  | 0.883 |
| (2 2 1) | -84325.95 | -84315.64 | -84279.73 | 165.3803 | 34.39045 | 41.78353 |  | 0.852 |
| (2 2 0) | -84403.68 | -84394.3 | -84361.65 | 176.9995 | 32.12119 | 40.43691 |  | 0.847 |
| (2 1 1) | -84323.08 | -84313.7 | -84281.06 | 165.9482 | 34.3901 | 41.69228 |  | 0.852 |
| (2 1 0) | -84401.19 | -84392.75 | -84363.37 | 174.5007 | 31.9166 | 40.78361 |  | 0.847 |
| (2 0 1) | -84677.67 | -84669.23 | -84639.85 | 157.2133 | 33.09954 | 41.52384 |  | 0.847 |
| (2 0 0) | -84765.25 | -84757.75 | -84731.63 | 165.4984 | 31.91586 | 38.59111 |  | 0.842 |
| (1 2 1) | -84331.94 | -84322.57 | -84289.92 | 166.0635 | 34.47144 | 41.37585 |  | 0.852 |
| (1 2 0) | -84409.29 | -84400.85 | -84371.47 | 164.2128 | 32.59549 | 40.31125 |  | 0.847 |
| (1 1 1) | -84329.71 | -84321.27 | -84291.89 | 164.3378 | 34.4786 | 41.48074 |  | 0.852 |
| (1 1 0) | -84407.43 | -84399.92 | -84373.81 | 160.9836 | 32.63961 | 40.35747 |  | 0.847 |
| (1 0 1) | -84684.23 | -84676.73 | -84650.61 | 149.1018 | 33.33941 | 41.62043 |  | 0.847 |
| (1 0 0) | -84771.84 | -84765.27 | -84742.42 | 157.1812 | 32.1007 | 38.80298 |  | 0.842 |
| (0 2 1) | -84422.87 | -84414.43 | -84385.05 | 176.6789 | 34.76623 | 41.93428 |  | 0.856 |
| (0 2 0) | -84503.13 | -84495.63 | -84469.52 | 151.9498 | 33.1524 | 41.82412 |  | 0.850 |
| (0 1 1) | -84420.48 | -84412.98 | -84386.87 | 172.5335 | 34.67713 | 42.71497 |  | 0.856 |
| (0 1 0) | -84501.25 | -84494.68 | -84471.83 | 154.4375 | 33.56019 | 40.95933 |  | 0.851 |
| (0 0 1) | -84791.6 | -84785.03 | -84762.18 | 175.5286 | 33.30737 | 42.25967 |  | 0.851 |
| (0 0 0) | -84880.72 | -84875.09 | -84855.5 | 173.5893 | 31.0533 | 41.90031 |  | 0.847 |
| (2 1 2 1) | -83217.18 | -83204.05 | -83158.35 | 277.2543 | 52.06433 | 21.61997 | 54.85966 | 0.814 |
| (2 1 2 0) | -83582.94 | -83571.69 | -83532.52 | 311.8581 | 57.42798 | 21.9349 | 45.14532 | 0.814 |
| (2 1 1 1) | -83215.87 | -83203.68 | -83161.25 | 275.4088 | 52.46804 | 21.42206 | 55.52562 | 0.814 |
| (2 1 1 0) | -83246.59 | -83235.34 | -83196.17 | 292.0994 | 51.08532 | 21.52848 | 54.84042 | 0.814 |
| (2 1 0 1) | -83380.68 | -83369.42 | -83330.25 | 310.0992 | 46.27643 | 20.92297 | 53.22689 | 0.808 |
| (2 1 0 0) | -83420.25 | -83409.93 | -40038.52 | 284.2138 | 46.87436 | 20.50927 | 54.04838 | 0.809 |
| (2 0 2 1) | -83540.22 | -83528.03 | -83485.59 | 296.6169 | 58.7478 | 22.77426 | 45.87915 | 0.816 |
| (2 0 2 0) | -83582.94 | -83571.69 | -83532.52 | 311.8581 | 57.42798 | 21.9349 | 45.14532 | 0.813 |
| (2 0 1 1) | -83539.99 | -83528.74 | -83489.57 | 295.5274 | 59.80041 | 22.81565 | 44.85728 | 0.816 |
| (2 0 1 0) | -83582.99 | -83572.68 | -83536.77 | 308.8228 | 56.85586 | 22.29575 | 43.95932 | 0.813 |
| (2 0 0 1) | -83796.22 | -83785.9 | -83750 | 260.7622 | 46.92346 | 21.29678 | 48.69336 | 0.808 |
| (2 0 0 0) | -83839.34 | -83829.97 | -83797.32 | 252.7566 | 46.13083 | 21.57928 | 46.85294 | 0.808 |
| (1 3 2 1) | -83230.61 | -83216.54 | -83167.58 | 320.9849 | 50.90145 | 21.82308 | 53.96646 | 0.814 |
| (1 3 2 0) | -83261.95 | -83248.82 | -83203.12 | 317.4979 | 51.31054 | 21.40194 | 54.73671 | 0.814 |
| (1 3 1 1) | -83229.03 | -83215.9 | -83170.2 | 315.46 | 51.95154 | 21.57857 | 54.20257 | 0.814 |
| (1 3 1 0) | -83260.15 | -83247.96 | -83205.52 | 319.4594 | 50.93839 | 21.44878 | 54.87911 | 0.814 |
| (1 3 0 1 ) | -83395.83 | -83383.64 | -83341.2 | 303.5374 | 46.95029 | 21.01659 | 53.12349 | 0.808 |
| (1 3 0 0) | -83435.66 | -83424.41 | -83385.24 | 301.2892 | 47.61323 | 20.29147 | 55.15603 | 0.808 |
| (1 2 0 3) | -83261.95 | -83248.82 | -83203.12 | 317.4963 | 21.40205 | 54.73631 | 51.31077 | 0.814 |
| (1 1 2 1) | -83233.98 | -83211.79 | -83169.35 | 316.2244 | 51.25872 | 21.87717 | 53.6124 | 0.814 |
| (1 1 2 0) | -83255.43 | -83244.17 | -83205 | 305.3123 | 51.04836 | 21.55053 | 54.5793 | 0.814 |
| (1 1 1 1) | -83222.7 | -83211.45 | -83172.28 | 319.2979 | 51.26236 | 21.70836 | 54.27661 | 0.814 |
| (1 1 1 0) | -83253.88 | -83243.57 | -83207.66 | 303.3165 | 50.95844 | 21.50233 | 55.19977 | 0.814 |
| (1 1 0 1) | -83389.74 | -83379.42 | -83343.52 | 297.7886 | 47.35037 | 21.07182 | 52.75476 | 0.808 |
| (1 1 0 0) | -83429.66 | -83420.28 | -83387.64 | 290.7062 | 47.88961 | 20.28261 | 55.62253 | 0.808 |
| (1 0 2 1) | -83545.07 | -83533.82 | -83494.65 | 311.3318 | 61.28712 | 22.62663 | 45.41689 | 0.816 |
| (1 0 2 0) | -83588.27 | -83577.96 | -83542.05 | 305.7383 | 58.74714 | 22.18828 | 44.79582 | 0.813 |
| (1 0 1 3) | -83395.83 | -83383.64 | -83341.2 | 303.5352 | 21.01676 | 53.1231 | 46.95038 | 0.808 |
| (1 0 1 1) | -83544.8 | -83534.49 | -83498.58 | 310.4133 | 60.40475 | 22.70901 | 45.48817 | 0.816 |
| (1 0 1 0) | -83588.3 | -83578.92 | -83546.28 | 302.7032 | 59.27946 | 22.4425 | 43.42655 | 0.813 |
| (1 0 0 3) | -83435.66 | -83424.41 | -83385.24 | 301.2791 | 20.29165 | 55.15539 | 47.61438 | 0.808 |
| (1 0 0 1) | -83804.2 | -83794.82 | -83762.18 | 282.5322 | 56.25917 | 19.55779 | 49.36193 | 0.808 |
| (1 0 0 0) | -83847.91 | -83839.47 | -83810.09 | 259.5641 | 50.17955 | 20.39705 | 48.82547 | 0.807 |
| (0 3 2 1） | -83275.63 | -83262.5 | -83216.8 | 336.0652 | 53.60278 | 21.62343 | 53.27803 | 0.816 |
| (0 3 2 0) | -83308.29 | -83296.1 | -83253.66 | 313.9009 | 53.66409 | 21.68451 | 51.96243 | 0.816 |
| (0 3 1 0) | -83306.46 | -83295.2 | -83256.03 | 315.4784 | 54.44957 | 21.25794 | 53.5608 | 0.816 |
| (0 3 0 1) | -83446.16 | -83434.9 | -83395.73 | 337.9335 | 47.1792 | 21.46388 | 51.93686 | 0.811 |
| (0 3 0 0) | -83486.94 | -83476.63 | -83440.72 | 336.5191 | 48.67146 | 21.5518 | 49.33776 | 0.811 |
| (0 2 1 3) | -83275.63 | -83262.5 | -83216.8 | 336.0486 | 21.6231 | 53.28109 | 53.60189 | 0.816 |
| (0 1 2 1) | -83268.58 | -83257.33 | -83218.16 | 345.9887 | 53.37697 | 21.68604 | 53.06144 | 0.817 |
| (0 1 2 0) | -83301.21 | -83290.89 | -83254.99 | 318.1174 | 54.16106 | 21.49926 | 52.45863 | 0.816 |
| (0 1 1 1) | -83267.32 | -83257 | -83221.09 | 334.0966 | 54.1736 | 21.51875 | 53.44138 | 0.817 |
| (0 1 1 0) | -83299.72 | -83290.34 | -83257.7 | 322.0692 | 54.62407 | 21.12138 | 54.08638 | 0.816 |
| (0 1 0 3) | -83306.46 | -83295.2 | -83256.03 | 315.4787 | 21.25801 | 53.56052 | 54.44985 | 0.816 |
| (0 1 0 1) | -83439.2 | -83429.82 | -83397.17 | 355.8159 | 47.42844 | 21.33953 | 52.05968 | 0.811 |
| (0 1 0 0) | -83480 | -83471.56 | -83442.18 | 330.179 | 48.38505 | 21.6388 | 49.48004 | 0.812 |
| (0 0 2 1) | -83588.13 | -83577.81 | -83541.9 | 312.6529 | 70.97437 | 24.17273 | 42.02973 | 0.822 |
| (0 0 2 0) | -83634.72 | -83625.34 | -83592.7 | 258.6594 | 68.24782 | 22.46207 | 44.46751 | 0.818 |
| (0 0 1 3) | -83446.16 | -83434.9 | -83395.73 | 337.9393 | 21.46404 | 51.93597 | 47.17955 | 0.811 |
| (0 0 1 1) | -83587.48 | -83578.1 | -83545.46 | 310.1588 | 72.7356 | 24.25564 | 41.63162 | 0.822 |
| (0 0 1 0) | -83634.55 | -83626.11 | -83596.73 | 283.1557 | 66.87563 | 22.6311 | 43.30954 | 0.818 |
| (0 0 0 3) | -83486.94 | -83476.63 | -83440.72 | 336.5133 | 21.55151 | 49.33896 | 48.67103 | 0.811 |
| (0 0 0 1) | -83861.42 | -83852.98 | -83823.6 | 330.7128 | 55.26252 | 21.32358 | 45.36686 | 0.812 |
| (0 0 0 0) | -83907.13 | -83899.63 | -83873.52 | 361.1884 | 57.97569 | 19.04528 | 51.66195 | 0.812 |

BIC: Bayesian Information Criterion; AIC: Akakie Information Criterion; OCC: Odds of correct classification

Table S4 Code of independent variables

| Variables Categories | Variables | Code |
| --- | --- | --- |
| Sociodemographic factors | Age | 0=60-74 years; 1=75-84 years; 2=85+ |
|  | Gender | 0=Male; 1=Female |
|  | Race | 0=White; 1=Black; 2=other race; 3=Hispanic |
|  | Marital status | 0=Partnered; 1=Not partnered |
|  | Education level | 0=College or higher (some college but no degree; associate’s degree; bachelor’s degree, master’s, professional, or doctoral); 1=Diploma level (vocational, technical, business, or trade school certificate or diploma); 2=High school (high school graduate); 3=Less than high school |
|  | Annual total income | 0 “>$6000”; 1 “$45000-60000”; 2 “$30000-44999”; 3 “$15000-29999”;  4 “<$15000” |
| Clinical factors | Body Mass Index (BMI) | 0 “Normal/non-obesity BMI<30.0”; 1 “obesity BMI≥30.0” |
|  | Self-rated health | 0=good; 1=fair; 2=poor |
|  | Smoke | 0=Yes;1=No |
|  | depression/anxiety | 0=normal; 1=mild depression/anxiety; 2=moderate depression/anxiety; 3=and severe depression/anxiety |
| Symptoms | Hearing impairment | 0= “No”; 1= “Yes” |
|  | Visual impairment | 0= “No”; 1= “Yes” |
|  | Swallowing impairment impairment | 0= “No”; 1= “Yes” |
|  | Speech impairment | 0= “No”; 1= “Yes” |
|  | Pain | 0= “No”; 1= “Yes” |
|  | Breathing problems | 0= “No”; 1= “Yes” |
